# Proteinuric Kidney Diseases: A Podocyte's Slit Diaphragm and Cytoskeleton Approach

**DOI:** 10.3389/fmed.2018.00221

**Published:** 2018-09-11

**Authors:** Samuel Mon-Wei Yu, Pitchaphon Nissaisorakarn, Irma Husain, Belinda Jim

**Affiliations:** ^1^Department of Medicine, Jacobi Medical Center, Bronx, NY, United States; ^2^Department of Medicine, James J. Peters VA Medical Center, Bronx, NY, United States; ^3^Renal Division, Jacobi Medical Center, Bronx, NY, United States

**Keywords:** cytoskeleton, nephrin, podocin, podocyte, slit diaphragm, synaptopodin, Rho/small GTPases, TRPC5/6

## Abstract

Proteinuric kidney diseases are a group of disorders with diverse pathological mechanisms associated with significant losses of protein in the urine. The glomerular filtration barrier (GFB), comprised of the three important layers, the fenestrated glomerular endothelium, the glomerular basement membrane (GBM), and the podocyte, dictates that disruption of any one of these structures should lead to proteinuric disease. Podocytes, in particular, have long been considered as the final gatekeeper of the GFB. This specialized visceral epithelial cell contains a complex framework of cytoskeletons forming foot processes and mediate important cell signaling to maintain podocyte health. In this review, we will focus on slit diaphragm proteins such as nephrin, podocin, TRPC6/5, as well as cytoskeletal proteins Rho/small GTPases and synaptopodin and their respective roles in participating in the pathogenesis of proteinuric kidney diseases. Furthermore, we will summarize the potential therapeutic options targeting the podocyte to treat this group of kidney diseases.

## Introduction

The kidney is dubbed the organ that filters out toxins and reabsorbs essential electrolytes from the blood to maintain homeostasis in our bodies. Podocytes, along with endothelial cells in the capillaries and glomerular basement membrane (GBM), form this filtration apparatus. The podocyte is a highly-differentiated cell that is composed of several structures: a cell body, primary process, secondary, and tertiary foot processes (FP) (1–7). Primary processes and foot processes (FPs) are highly supported by a complex cytoskeletal architecture, which in of itself is intertwined by a network of microtubules, intermediate filaments, and actin (8, 9). This complex network (microtubules, intermediate filaments, and actin) not only accommodate podocytes to the constantly changing environment, they are crucial hubs of signal transduction within podocytes (10). Furthermore, the neighboring FPs form an interdigitating structure lining outside of the capillary walls (11) and are connected by a specialized cell junction called the slit diaphragm (SD) (12–15). The SD and the negatively charged surface on FP restrict protein from filtering through. Either a dysregulated podocyte cytoskeleton or the loss of integrity of the SD can result in podocyte foot process effacement (FPE), the most common pathological finding in podocyte diseases. Proteinuria is, therefore, the pathognomonic phenomenon in podocytopathy (16). Research on podocyte pathophysiology is crucial due to their function and their lack of ability to regenerate, despite more recent data supporting its potential regenerative capacity (17). In this review, we will summarize our current understanding of podocyte signaling pathways via the SD proteins and the actin cytoskeleton and how they cause proteinuric diseases. We will also discuss the latest updates in using the podocyte as a target for drug development.

### Slit diaphragm proteins and disease implications-nephrin, podocin, and TRPC

**Figure 1:**
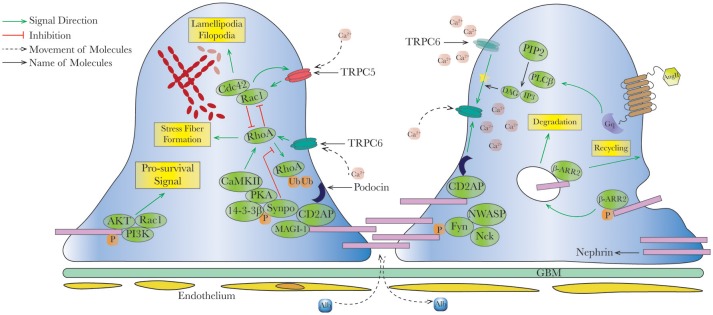
Schematic interaction between slit diaphragm signal cascades and actin dynamics in healthy podocytes. In healthy podocytes, slit diaphragm (SD) is composed by various proteins and acts as a signaling hub, to maintain podocyte survival and regulated actin dynamics. For example, nephrin phosphorylation recruits AKT/PI3K and Nck/NWASP to modulate pro-survival signaling and actin organization, respectively, in concert with other SD proteins such as podocin, CD2AP, and synaptopodin. In physiological status, predominant TRPC6 offers necessary intracellular calcium and activates RhoA, an important small GTPase promoting stress fiber formation. Imbalance between RhoA and Rac1/Cdc42 (activated by TRPC5) leads to dysregulated actin formation. Upon stimulation (for example angiotensin II), G-protein coupled receptors (GPCRs) triggers second messengers' formations, further modulating intracellular calcium concentration via activating TRPC6 (yellow star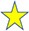). Endocytosis and recycling of proteins on slit diaphragm is controlled by serial phosphorylation and associated proteins such as β-arrestin2. PI3K, phosphatidylinositol-4,5-bisphosphate 3-kinase; Rac1, ras-related C3 botulinum toxin substrate 1; Cdc42, cell division control protein 42 homolog; RhoA, ras homolog gene family, member A; CaMKII, ca^2+^/calmodulin-dependent protein kinase II; Synpo, synaptopodin; MAGI-1, membrane-associated guanylate kinase inverted-1; PIP2, phosphatidylinositol bisphosphate; PLCβ, phospholipase Cβ; DAG, diacylglycerol; IP3, inositol 1,4,5-trisphosphate; β-ARR2, β-arrestin 2.

In the past two decades, an increasing number of disease-causing SD proteins has been identified (5). During kidney development, the SD initially incorporates the features of cell-cell junction which eventually matures into a zipper-like structure by molecular cross-linking via proteins such as nephrin, podocin, and Neph1. Other SD-related proteins may either participate in junctional formation (Nck, CD2AP) or not (TRPC). It is now known that the SD not only acts as a filter to prevent proteinuria (18), but serves as a complex signaling hub to divert different chemical and mechanical stimuli to podocytes (19). We will highlight the best-studied proteins on the SD with clinical significance: nephrin, podocin, and transient receptor potential canonical (TRPC) proteins (Figure 1).

#### Nephrin

Most of our understanding of nephrin stems from the successful cloning from patients with Congenital Nephrotic Syndrome of the Finnish type (CNF) (20). Nephrin, a 180-kDa protein encoded by *NPHS1*, consists of a cytoplasmic C-terminus, a short transmembrane domain, and an extracellular fibronectin type III motif attached with eight distal IgG-like motifs (19, 21). It acts as an intracellular signaling scaffold (22–24) and a podocyte cytoskeleton modulator (25–27). Consistent with the high mortality in children born with CNF, global nephrin-null mice died soon after birth. The technical difficulties of studying its biological function (28, 29) were overcome by the discovery of podocyte-specific promoters (30–33) and the use of short hairpin RNA (34). Of note, although nephrin-null mice are not compatible with life, more than 80–90% successful knockdown of nephrin only resulted in mild proteinuria unless second renal injuries were implemented, suggesting more pivotal role of nephrin in prenatal slit diaphragm development (34). Most of the nephrin signaling occurs via the cytoplasmic tail that is enriched by the highly-conserved tyrosine residues (35) which are phosphorylated by a series of Src-family kinases such as Fyn (23, 26, 36–38). Verma et al. (26) and Jones et al. (27) established the concept of a nephrin-Nck complex as an adaptor after nephrin phosphorylation to regulate actin reorganization/polymerization (39–46), in concert with other signaling pathways such as PI3K/Akt/Rac1 (25, 47, 48).

To date, derangement of nephrin can be largely divided into four mechanisms: (1) decreased phosphorylation, (2) increased endocytosis, (3) loss of function due to genetic mutations, and (4) downregulation at both the mRNA and protein level. This loss of the nephrin-led signaling cascade eventually causes the proteinuric phenotype (27, 49, 50) Nephrin dephosphorylation can be induced by angiotensin II (AngII) which results in the diminished interaction with c-Abl and Akt inactivation, pathways that promote cell survival and growth (51); in addition, it can be caused by the c-maf inducing protein (c-mip) which inhibits interactions between Fyn and the cytoskeletal regulator N-WASP as well as between the adaptor protein Nck and nephrin, resulting in actin cytoskeleton disorganization and foot process effacement (52). Decreased nephrin phosphorylation caused by phosphatases were also observed in various models (53), such as protein tyrosine phosphatase 1B (PTP1B) in rat with puromycin aminonucleoside nephrosis (PAN) (54) and Src homology region 2 domain-containing phosphatase-1 (SHP-1) in human podocytes with hyperglycemia (55). The blockade of phosphatases on nephrin may thus be a therapeutic target in proteinuric diseases and has shown positive results in murine models (lipopolysaccharide[LPS]-induced proteinuria) (56, 57). Nephrin endocytosis through β-arrestin can be stimulated by AngII (58). and hyperglycemia, the latter effect occurs via the interaction of β-arrestin2 with PKC-α which can be abrogated by knocking down PKC-α (59, 60). Interestingly, the loss of phosphorylation of nephrin increases nephrin endocytosis via β-arrestin2 (61), suggesting that nephrin is under a location-specific regulation. Apart from the original mutation described in CNF, there are presently almost 200 disease-associated *NPHS1* mutations that have been described (62–66). The phenotypic variability of these mutations has shown us that the nephrotic syndrome manifests not only in the first 90 days of life, but can occur much later on in life as well (67). Furthermore, compound heterozygous or homozygous *NPHS1* mutations have also been identified in families with adults with focal segmental glomerulosclerosis (FSGS) (65). In addition to mutations, single nucleotide polymorphisms (SNPs) in NPHS1 and WT-1 have also been identified to confer risk end-stage kidney disease in African Americans (68) and FSGS (69), respectively. Nephrin expression is further regulated by transcription factors such as WT1 (70, 71), NF-κB (72), Sp1 (specificity protein 1) (73), Furthermore, studies have demonstrated that *Wilms' tumor 1* (*WT1*) not only functions as a pivotal gene in kidney development, it also regulates other important SD proteins by binding to the proximal promoters, such as *Nphs2, Synaptopodin*, and *Nck2* (74, 75). Downregulation of nephrin has been observed in many human glomerular diseases by detecting protein and mRNA levels, such as minimal change disease (76), membranous nephropathy, membranoproliferative glomerulonephritis, IgA nephropathy, lupus nephritis (77), diabetic kidney disease (DKD) (78, 79), and preeclampsia (80). Detection of urinary nephrin protein (nephrinuria) or mRNA has already been studied as an early biomarker of disease in DKD, glomerulonephritis, and preeclampsia (81–87) with variable success.

#### Podocin

After successful cloning of *NPHS1*, a new mutated gene *NPHS2*, which encodes for podocin on chromosome 1q25-31, was mapped in patients with autosomal-recessive steroid-resistant nephrotic syndrome (SRNS) (88, 89). Patients' clinical presentations can range from early-onset nephrotic syndrome (typically from 3 months to 6 years of life), FSGS, to end-stage kidney disease (ESKD) by the first decade of life (90, 91). Podocin is a hairpin-like protein including both cytoplasmic N- and C-terminus with a transmembrane portion (92). Almost exclusively expressed on SD of the kidneys (93), podocin interacts with phosho-nephrin and Neph1, another SD protein with a similar structure to nephrin; the podocin-Neph1 interaction is believed to be important for recruitment of nephrin to the lipid raft which augments nephrin signaling (23, 94–99). The prohibin homology (PHB) domain of podocin does not participate in hairpin formation or mediate podocin endocytosis (100), though it may regulate the activity of the TRPC6 channel and act as a mechanic sensor in podocytes (101–103). Among the several types of mutations, nonsense and missense mutations of *NPHS2* fail to recruit nephrin into raft domains (94). Other types of nephrotic syndrome (NS), including familial and sporadic steroid resistant nephrotic syndrome (SRNS) (28–40% and 6–19%, respectively) (90, 91, 104–107), adult-onset FSGS (108–110), and non-diabetic ESKD in African Americans (111) have also found to exhibit *NPHS2* mutations. The type of mutation, whether heterozygous or homozygous, appears to correlate with a different phenotype. For example, patients with two pathogenic NPHS2 mutations have presented with an early onset of SNRS with a low incidence of post-transplant recurrence, whereas heterozygous *NPHS2* variants seem to exhibit a milder, late-onset phenotype with a higher incidence of recurrence (91, 105, 112). Furthermore, mutations of the transcription factors LMX1B and FoxC (113–115) that control podocin expression can also result in disease, such as the nail-patella syndrome or isolated hereditary FSGS (116, 117). Interestingly, the transcription factor WT1 can also regulate LMX1B and FoxC expression, adding an extra layer of modulation in podocin expression (118). Currently, more than 1,000 podocin variants that have been described (https://databases.lovd.nl/shared/genes/NPHS2) (119) and further study will be needed to understand their individual roles, particularly in different genetic backgrounds (120). Lastly, podocin downregulation has also been observed in lupus nephritis (121), pediatric nephrotic syndrome (122), and FSGS (123), but not in preeclampsia (80).

#### TRPC

Transient receptor potential canonical (TRPC) channels are a group of seven families of non-selective cationic channels (TRPC1 to TRPC7) and largely divided into TRPC3/6/7 and TRPC1/4/5 based on sequence alignment and functionality (124). Among them, TRPC6 and TRPC5 have been identified in podocytes and have been widely researched in the past decade. The impetus for this research started after a gain-of-function mutation was identified in a family of autosomal FSGS (125, 126). Like other TRPCs, TRPC6 can be activated by phospholipase C (PLC)-dependent pathway via the generation of diacylglycerol (DAG) and inositol 1,4,5-phosphate (IP3), resulting in the subsequent Ca^2+^ influx into podocytes. Winn et al. demonstrated that a gain-of-function mutation in TRPC6 (proline to glutamine substitution at position 112) enhanced calcium signaling via AngII stimulation and cell surface expression of TRPC6 in HEK 293 cells (126), which ultimately causes eventual podocyte apoptosis and detachment (127). Recently, a TRPC6 loss-of-function type of mutation was also found to be associated with human FSGS (128), thus providing evidence that reduced *physiological* TRPC6-mediated calcium entry may also cause disease. In addition to FSGS, TRPC6 has been reported to affect DKD by increasing TRPC6 expression mediated by AngII as seen in streptozotocin-induced diabetic rats; this suggests a role for TRPC6 in causing podoctyopenia and proteinuria (129, 130). TRPC5 and TRPC6 both affect the actin cytoskeleton differently, i.e., upon activation through AngII, TRPC6 couples with Rho A (see section on Rho/Small GTPases) while TRPC5 couples with Rac1, resulting in stress fiber vs. motile phenotype in the podocyte, respectively. TRPC5-mediated signals have been considered as “disease-type” leading to cytoskeletal collapse (131). The function of TRPC5, however, has recently been put in question. There is evidence that deletion of TRPC5, either genetically or pharmacologically, results in decreased glomerular injury when exposed to LPS or protamine sulfate (PS) (132). Recently, Zhou et al. showed that inhibition of TRPC5 with a small molecule inhibitor (AC1903) prevented podocyte loss in a rat model of AT1R overexpression (model of FSGS) and halted the disease progression in the Dahl salt-sensitive rate model of hypertensive kidney disease (133). These findings, however, were contradicted by another study suggesting that the overexpression TRPC5 in mice does *not* cause proteinuria nor aggravate LPS-induced albuminuria (134). Nonetheless, these conflicting findings further complicate our current understanding of TRPC channelopathy on podocytes, and in fact, echo some of the contradictory results in GTPases studies in this field (see below).

### Podocyte signaling at actin cytoskeletons- Rho/Small GTPases, and synaptopodin

Derangements in the actin cytoskeleton of podocytes have been widely reported to cause proteinuric kidney diseases. Not only does it maintain the structural integrity of the podocytes, but it also mediates complex signal cascades, endocytic pathways, and interactions outside of podocytes such as the GBM (10). Here we will focus on Rho-family of small GTPases and synaptopodin.

#### GTPases

The Rho-family of small GTPases such as RhoA, Cdc42, and Rac1 regulate actin cytoskeleton remodeling by acting as molecular switches to coordinate signaling by either promoting cell motility or inhibiting cell migration (135). Each GTPase cycles between an active GTP-bound state and an inactive GDP-bound state. Upon activation, Rho GTPases are able to bind to a range of effector proteins to regulate downstream signaling pathways. Rho-dependent pathways are activated by AngII, platelet-derived growth factor, endothelin-1 (136), and promote contractile phenotype with the formation of stress fibers. On the contrary, Cdc42/Rac1 activation leads to lamellipodia/filopodia formation, an unstable phenotype of podocytes which causes FPE (137). Although initially it seemed that RhoA activation with Cdc42/Rac1 suppression was beneficial to the podocyte, studies in the past decade have shown conflicting results. For example, transgenic mice with constitutive activation of RhoA in podocytes resulted in proteinuria with FPE, podocyte apoptosis, decreasing nephrin expression (138) and histologic features of FSGS (139). Inhibiting RhoA signaling was thus shown to mitigate podocyte injury murine chronic kidney disease model (5/6 nephrectomy) and rats with PAN (140, 141). However, constitutive expression of dominant-negative RhoA in mice also caused proteinuria and loss of stress fiber formation in mouse podocytes (138), while preserving RhoA signaling in mice with LPS-induced proteinuria via synaptopodin (142) showed anti-proteinuric effects (143). Thus, the balance of RhoA signaling is imperative for podocyte, and it is likely to be dependent on the specific disease state. In the Cdc42/Rac1 system, podocyte-specific Rac1 knockout mice were shown to exhibit normal podocyte morphology and protection from PS induced-podocyte injury (144). However, these Rac1 knockout mice were also found to exacerbate the injury from chronic hypertensive glomerulosclerosis. Activation of Rac1 has shown to attenuate podocyte injury and expedite recovery *in vitro* (145, 146), yet hyperactivation of Rac1 has been linked to diseased podocytes in a dose-dependent manner (147–149). For Cdc42, podocyte-specific deletion in mice led to early-onset severe proteinuria, FPE, glomerulosclerosis (144), and congenital nephropathy (150). Similarly, reduced Cdc42 expression resulted in mouse podocyte apoptosis due to downregulated Yes-Associated Protein (YAP) signaling (151, 152) and promoted filopodia formation. Decreased activity of both Cdc42 and Rac1 via *FAT1* mutation has shown to cause human and mouse glomerulotubular nephropathy (153). These results collectively suggest Rac1 signaling might be dispensable for podocyte development as compared with Cdc42 (150), but whether Rac1 deletion is beneficial or detrimental, again, depends on the type of podocyte injury.

Mutations in human genes that interfere with Rho GTPase signaling have been identified in nephrotic syndrome (154), FSGS (155), and DKD (156). For example, mutations in *ARHGDIA*, which exists in a complex with Rho GTPases, increased levels of Rac1 and Cdc42 (but not RhoA) have been found in individuals with SRNS (Table 1) (154). The human disease mutation in Rac1-GTPase-activating protein (Rac1-GAP) *ARHGAP24*, which results in elevated Rac1 and CDC42 levels, was also found to be associated with a family with FSGS (155). Hence, we are only beginning to learn more about the significance of these subclasses of the Rho-family of small GTPases as they appear to have divergent roles in specific disease states.

**Table 1:** Genes of slit diaphragm and actin cytoskeleton that are associated with proteinuric kidney diseases.

	**Diabetic nephropathy**	**Focal segmental glomerulosclerosis**	**Membranous nephropathy**	**Minimal change disease**
Slit diaphragm	•*TRPC6* (130) •*NPHS1*(78, 157–159) •*NPHS2* (159)	•*NPHS1* (20, 65) •*NPHS2* (89–91, 105, 108–110, 160) •*CD2AP* (161–163) •*TRPC6* (126, 128, 164–168) •*PLCE1* (169)	•*NPHS1* (170) •*NPHS2* (171)	•*NPHS1* (76, 172, 173) •*NPHS2* (119, 174)
Actin cytoskeleton	•*Rac1* (175) •*PTEN* (156)	•*ACTN4* (176–178) •*INF2* (179–181) •*FAT1* (153) •*MYO1E* (182) •*ARHGP24* (155) •*ARGHDIA* (154) •*Rhpn1* (183) •*YAP* (184)	•*C-MIP* (185) •*CRK1/2* (186)	•*KANK* (187) •*CFL1* (188) •*C-MIP* (185)

*FPE, foot process effacement; TRPC, Transient receptor potential canonical; PTEN, phosphatase and tensin homolog; CD2AP, CD2-associated protein; PLCE1, phospholipase C epsilon 1; ACTN4, alpha-actinin 4; INF2, inverted formin 2; FAT1, fat cadherin 1; MYO1E, myosin IE; AHRGP24, Rho GTPase activating protein 24; ARGHDIA, Rho-GDP dissociation inhibitor alpha; YAP, yes-associated protein; C-MIP, c-maf-inducing protein; KANK, KN motif and ankyrin repeat domain; CFL1, cofilin-1*.

#### Synaptopodin

Synaptopodin is a proline-rich actin-binding protein highly expressed in dynamic cells such as telencephalic dendrites and podocytes. In podocytes, synaptopodin was found to interact with α-actinin-4, an important protein to cross-link between actin filaments (189). Loss of synaptopodin led to delayed stress fiber formation and development of aberrant nonpolarized filopodia. Although the ultrastructure of podocytes in synaptopodin-deficient (synpo^−/−^) mice appeared to be normal, these mice showed impaired recovery from both PS- and LPS-induced kidney injury (190). Synaptopodin was later revealed to block Smurf1-mediated ubiquitination of RhoA to preserve stress fibers formation by increasing RhoA expression and activation (142). By contrast, synaptopodin inhibits binding of Cdc42 and Mena to IRSp53 and therefore protects aberrant filopodia formation in mouse podocytes (191). Synaptopodin is phosphorylated by PKA/CaMKII and binds 14-3-3ß to promote Rho signaling (143). Under TRPC5-mediated calcium influx, synaptopodin is degraded by calcineurin-mediated phosphorylation and promotes Rac1 signaling and ROS formation (192). Cyclosporine A, a calcineurin inhibitor used in immunosuppression, was found to have an anti-proteinuric effect due to blocking calcineurin-mediated dephosphorylation of synaptopodin (143). Additionally, Yu et al. demonstrated that synaptopodin affects TRPC6 localization and function which suggests that decreased synaptopodin levels under diseased conditions will increase TRPC6-mediated calcium influx and induce podocyte apoptosis (193). Clinically, reduced expression of synaptopodin has been observed in FSGS (176, 194), HIV-associated nephropathy (HIVAN) (194–196), IgA nephropathy (197), and idiopathic nephrotic syndrome of childhood (198). Synaptopodin levels have been shown to correlate with disease severity and response to treatment in human FSGS and minimal change disease (MCD) (199, 200). Urinary synaptopodin protein and mRNA excretion have also been used to assess the decline of renal function and certain glomerular diseases (201, 202).

### Clinical implications

**Figure 2:**
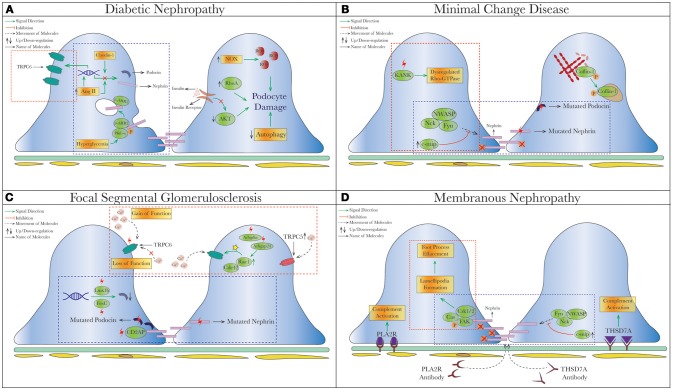
Schematic depiction of pathophysiologic processes in specific proteinuric kidney diseases. Dysregulated slit diaphragm and altered actin dynamics depicted in specific disease states that cause podocyte foot process effacement (FPE) and podocyte loss. The commonalities of pathophysiology in different diseases are grouped into blue 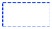 (dysregulated pathways related to SD proteins) or red 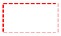 (cytoskeletal dysregulation via TRPC or Rho-GTPase) dashed square. **(A)** Diabetic Nephropathy. Under hyperglycemia, nephrin endocytosis can be induced by the interaction of β-arrestin2 with PKC-α (59, 60). Increased AngII (78, 158) and Claudin-1 (159, 203) in diabetes were both shown to reduce expression of nephrin and podocin. On the contrary, increased TRPC6 expression mediated by AngII was seen in streptozotocin-induced diabetic rats causing podocytopenia and proteinuria (129, 130). Hyperglycemia also leads to podocyte apoptosis from production of reactive oxygen species (ROS) through NAPDH oxidase (NOX) (204), so as does insulin resistance due to reduced AKT pro-survival signaling (205). **(B)** Minimal Change Disease. Mutations of nephrin (*NPHS1*) and podocin (*NPHS2*) have been reported in MCD and tended to have higher rates of steroid-resistance (119, 174, 206), as well as recessive mutations of KANK (kidney ankyrin repeating-containing protein) *KANK1* and *KANK2* identified in a cohort of Arab and European origins (187). Cofilin-1, actin-binding protein necessary for maintaining podocyte architecture (207), is inactivated by phosphorylation seen in human MCD, leading to its redistribution to nucleus in the disease states (188). A novel molecule c-mip noted to be upregulated in human MN/MCD was shown to impair podocyte actin reorganization by inhibiting interaction between Fyn/N-WASP and nephrin/Nck. Podocyte overexpressed with c-mip could result in downregulation of nephrin and synaptopodin (52, 185, 208). **(C)** Focal Segmental Glomerulosclerosis (FSGS). Multiple gene mutations have been identified in patients with FSGS through genetic studies. Most of them encode various critical podocyte structures or signaling pathways, such as SD complex (*NPHS1, NPHS2, CD2AP)*, SD-related Ca^2+^ signaling (*TRPC6, PLCE1*), actin cytoskeleton/endocytosis *(ACTN4, INF2, FAT1, MYO1E)*, and small-GTPases (*AHRGP24, ARGHDIA, ARHGEF17*) (209–214). A novel mechanism of increased podocin endocytosis via sorting nexin 9 (SNX9), which is seen in human IgA nephropathy, membranous nephropathy and FSGS was also described (171). Blocking the imbalanced TRPC5/6 signaling and increased circulating permeability factors (suPAR/CLC-1) are possible new therapeutic approaches in FSGS. **(D)** Membranous Nephropathy. Besides the well-known antibody-mediated primary MN (anti-PLA2R/THSd7A), reduced nephrin phosphorylation (170) and increased podocin endocytosis (171) have been described in human MN. Disorganization of actin cytoskeleton from a different cytoskeletal pathway Cas-FAK-Crk1/2 activation (not the typical nephrin-Nck pathway) was also seen in human MCD and MN (186). Mutation (red thunder 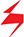) of proteins, inhibiting (red cross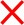) or activating (yellow star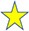) different signal pathways leads to reduced level of phosphorylation, increased intracellular calcium and endocytic process. AngII, Angiotensin II; c-ABL, Abelson murine leukemia viral oncogene homolog 1; RhoA, ras homolog gene family, member A; NOX, NAPDH oxidase; ROS, reactive oxygen species; Cdc42, cell division control protein 42 homolog; Rac1, ras-related C3 botulinum toxin substrate 1; Lmx1b, LIM homeobox transcription factor 1-beta; SNX-9, sorting nexin 9; suPAR, soluble urokinase plasminogen activator receptor; CLC-1, cardiotropin-like cytokine-1; FAK, focal adhesion kinase; PLA2R, phospholipase A2 receptor; THSD7A, thrombospondin Type 1 domain containing 7A; c-mip, c-maf-inducing protein; KANK, kidney ankyrin repeating-containing protein.

Advances in our understanding of podocyte biology have been a great asset to elucidate underlying pathophysiology in proteinuric kidney diseases. DKD, MCD, FSGS, and membranous nephropathy (MN) were all found to have dysfunction at the SD or actin cytoskeletal system (Figure 2). Although the therapeutic options targeted to podocytes are limited, the concept of restoring SD proteins, stabilizing the cytoskeleton, or stimulating podocyte regeneration is showing promise.

#### Renin-angiotensin-aldosterone system inhibition

Inhibiting AngII has shown to be beneficial in several models of glomerular diseases. In DKD, there is a large body of evidence that shows inhibition of AngII results in the reduction in proteinuria and the slowing of the rate glomerular filtration rate (GFR) decline in humans. In addition to the well-known hemodynamic effects of inhibiting the renin-angiotensin-aldosterone system (RAAS) (215), both angiotensin-converting enzyme inhibitor (ACEI) and AngII type 1 receptor blocker (ARB) have shown to significantly increase nephrin expression in murine diabetic models (157, 216). A similar effect was also observed in rats models by adriamycin-induced nephropathy and PAN (217, 218). Since AngII has a direct TRPC6-mediated calcium signaling (219, 220) which results in ROS expression (221) and proteinuria (222), inhibiting TRPC6 indirectly via AngII blockade is a feasible strategy in proteinuric diseases such as FSGS.

As described above, the concept of podocyte's limited capacity of regeneration has been challenged by novel genetic labeling techniques to trace possible podocyte progenitors. The exact origin of the detected newly-developed podocytes is still under debate; to name a few, glomerular parietal epithelial cells (PEC) and cells of renin lineage (CoRL) have been most investigated to date. Surprisingly, RAAS inhibition has been also shown to increase podocyte numbers either from PEC (223, 224) or CoRL (225, 226), suggesting its unique property and potential therapeutic effect.

#### Glucocorticoid

Glucocorticoid (GC) is the backbone and initial therapy for several nephrotic syndrome including FSGS (209) and MCD (227). In addition to the effects on immunomodulatory cells, cultured murine podocyte was shown to carry glucocorticoid receptor (GR) complex and respond to GC in a dose- and time-dependent manners at both transcriptional and posttranscriptional levels (228). In an immortalized human podocyte model, the expression of nephrin and other podocyte genes was upregulated after dexamethasone treatment (229, 230). GC was also shown to help to stabilize the actin cytoskeleton system (231), and to increase actin stress fiber formation via overexpression of Krüppel-like factor 15, a downstream target of GC (232). Lastly, in murine model of experimental FSGS (via cytotoxic anti-podocyte antibody), GC might regenerate podocytes from adjacent PECs (233). Therefore, the therapeutic actions of GC in proteinuric diseases may very well be due to its myriad benefits on the podocyte (234).

#### Inhibition of Rho-associated kinases (ROCKs)

With murine diabetic and PAN models, inhibiting downstream effectors Rho-associated kinases (ROCKs) using ROCK inhibitors has shown renoprotective effects (235, 236). Increased Rac1 and mineralocorticoid receptor signaling were also found to be increased in mice lacking Rho GDP-dissociation inhibitor-alpha [*Arhgdia*(^−/−^) mice] with heavy albuminuria and podocyte damage. A Rac-specific small-molecule (NSC23766) and eplerenone (mineralocorticoid receptor antagonist) have successfully ameliorated the renal damages seen in *Arhgdia*(^−/−^) mice, suggesting the therapeutic effects by suppressing Rac1 or mineralocorticoid receptor signaling (237). Nevertheless, conflicting data in this field demands additional meticulous studies to further illuminate future therapeutic targets.

#### Vitamin D and retinoic acids

Selective vitamin D receptor activation was proven to lower proteinuria in patients with DKD (238); it was later demonstrated that vitamin D3 analogs might ameliorate podocyte injury by reversing the decrease in nephrin expression induced by hyperglycemia (239–241). The combination of vitamin D analog with an ARB has been shown to have synergetic effects on RAAS blockade, thereby boosting its overall therapeutic effect (242, 243). However, as there is currently no recommended clinical dosage of vitamin D analog for use in DKD, long-term and prospective clinical data is required to assess its safety and efficacy (244, 245). Retinoic acids (RAs) are vitamin A derivatives and appear to have renoprotective effects on various murine nephropathy such as HIV-associated nephropathy (HIVAN) (246) and PAN-induced nephrotic rats (247). Recently, Dai et al. reported that RA treatment activated podocyte retinoic acid receptor-α (RAR-α) and increased synaptopodin expression on PEC in wild type mice with nephrotoxic serum-induced glomerulonephritis (GN). The group subsequently identified RAR-α expression in human crescentic GN kidney biopsies, substantiating a possible therapeutic option in treating human crescentic GN (248). RA was also found to induce podocyte markers in PECs in a murine model membranous nephropathy (Heymann nephritis) and FSGS (anti-glomerular antibody model) (249). However, the toxicities of RA have hindered it from wide clinical use and have resulted in difficulties in recruiting patients to a Phase II clinical trial of podocyte diseases (MCD, FSGS, collapsing glomerulopathy) (NCT 00098020) (250). Thus, less toxic alternatives, such as Kruppel-like factor-15 and boronic acid retinoid are being explored (250, 251).

#### Biological agents

Emerging evidence also suggests that biological agents might regulate the podocyte SD. Rituximab (RTX), an anti-CD20 antibody which depletes B cells, is widely used in different proteinuric diseases such as FSGS (252–254) and steroid-resistant MCD (255–258). Besides its immunological response, RTX was found to preserve sphingomyelin-phosphodiesterase-acid-like 3b (SMPDL-3b), which in turn regulates acid sphingomyelinase (ASMase) activity in the raft of podocytes (259). SMPDL-3b can partially co-localize with synaptopodin and hence prevent the actin remodeling that occurs after exposure to serum from FSGS patients (259). This concept was supported by a recent clinical observation of the increase in urinary SMPDL-3b in patients at the remission stage after RTX treatment compared with the proteinuric stage (260). Abatacept (CTLA-4-Ig), a costimulatory-inhibitor that targets B7-1 molecule, may be another therapeutic agent to treat nephrotic syndrome via the actin cytoskeleton. Since induction of the T-cell costimulatory molecule B7-1 by LPS has been shown to be associated with nephrotic syndrome and actin reorganization (261), an inhibitor of B7-1 may ameliorate these findings. Though Yu et al. initially reported that treating five FSGS patients with abatacept achieved partial remission (262), these findings have not been successfully replicated (263–266). Presently, there is an ongoing phase II trial of abatacept for patients with FSGS or MCD (NCT02592798).

## Conclusion

Our improved understanding of the podocyte in terms of its SDs and cytoskeleton has allowed us to target this cell for new treatments for proteinuric diseases (267, 268). However, there is much more to discover. Novel therapeutic target genes or proteins in podocytes are constantly being discovered as we write this review (269–271). Longstanding concepts, such as the function of the SD and the lack of mitotic ability of the podocyte, are already being challenged (272–274). For instance, the use of glycogen synthase kinases 3 inhibitor has already demonstrated initial promising results of transforming renal progenitor cells into podocytes (275). Growing podocytes from induced pluripotent stem cells on the chip also serves as a great tool to assess new drug development (276). At the same time, newer imaging systems to count podocytes (277) and to study their membrane dynamics have enhanced our ability to study this cell (278). The information that we hope to garner from the field of “-omics” (279, 280) reminds us that data is both tremendously helpful and potentially fraught with error, and hence must be interpreted with great care (281, 282). Thus, persistent endeavors to elucidate complex podocyte biology (283, 284), along with long-term patient follow-up (285), would greatly add to our present understanding of these common yet poorly understood proteinuric diseases.

## Author contributions

SY, PN, IH, and BJ wrote draft of the manuscript. SY contributed to the figures. SY and BJ reviewed and edited the manuscript before submission.

### Conflict of interest statement

The authors declare that the research was conducted in the absence of any commercial or financial relationships that could be construed as a potential conflict of interest.
